# The annual cost of not breastfeeding in Indonesia: the economic burden of treating diarrhea and respiratory disease among children (< 24mo) due to not breastfeeding according to recommendation

**DOI:** 10.1186/s13006-018-0152-2

**Published:** 2018-03-02

**Authors:** Adiatma Y. M. Siregar, Pipit Pitriyan, Dylan Walters

**Affiliations:** 1 Center for Economics and Development Studies, Department of Economics, Faculty of Economics and Business, Universitas Padjadjaran, Jl. Cimandiri no. 6-8, Bandung, West Java 40115 Indonesia; 2 Canadian Centre for Health Economics, Institute of Health Policy, Management and Evaluation, University of Toronto, Toronto, Ontario Canada

**Keywords:** Breastfeeding, Cost of not breastfeeding according to recommendation, Inadequate breastfeeding, Suboptimal breastfeeding, Economic analysis, Indonesia

## Abstract

**Background:**

In Indonesia, 96% of children (< 24mo) are breastfed. However, only 42% of children (< 6mo) are exclusively breastfed, as per World Health Organization recommendations. Breastfeeding provides protective benefits such as reducing the risk of morbidity and mortality associated with diarrhea and pneumonia/respiratory disease (PRD). This study estimates the potential economic impact of not breastfeeding according to recommendation in Indonesia based on infants suffering from attributable diarrhea and PRD.

**Methods:**

A cost analysis examined both the healthcare system costs and non-medical costs for children (< 24mo) with diarrhea and PRD. Data collection took place between 2015 and 2016 and healthcare expenditures were assessed in 13 facilities, in five sites including Bandung and Tomohon City. Costs from a provider perspective were estimated using healthcare records and 26 interviews with healthcare workers. A discount rate of 3% was used. A cross-sectional survey with caregiver-child pairs (*n* = 615) collected data related to out of pocket costs such transportation and opportunity costs such as wage loss. These figures were combined with the national disease prevalence rates from Indonesia Demographic and Health Survey 2012, and the relative risk of disease of not breastfeeding according to recommendation from literatures to extrapolate the financial burden of treatment.

**Results:**

The healthcare system cost due to not breastfeeding according to recommendation was estimated at US$118 million annually. The mean healthcare system cost and out of pocket costs was US$11.37 and US$3.85 respectively. This cost consists of US$88.64 million of provider costs and US$29.98 million of non-medical patient costs.

**Conclusions:**

The cost of not breastfeeding according to recommendation is potentially high, therefore the Indonesian government needs to invest in breastfeeding protection, promotion and support as the potential healthcare system cost savings are significant. As suggested by other studies, the long term cost due to cognitive losses of providing not breastfeeding according to recommendation should also be taken into account to provide a complete understanding of the economic impact of not breastfeeding according to recommendation.

**Electronic supplementary material:**

The online version of this article (10.1186/s13006-018-0152-2) contains supplementary material, which is available to authorized users.

## Background

Breastfeeding has been shown to provide many benefits to children, mothers, health systems and economies. Breastfeeding is associated with decreasing maternal risk of breast cancer, ovarian cancer and Type 2 diabetes, and higher intelligence of the child and decreased risks of infections, malocclusion of the teeth, overweight and diabetes [1, 2]. Breastfeeding according to recommendation consists of early initiation of breastfeeding (within the first hour of birth), exclusive breastfeeding for the first six months, and continued age appropriate breastfeeding at two years (accompanied by iron-rich complementary foods) [3].

Not breastfeeding according to recommendation is associated with diarrhea and pneumonia/respiratory disease (PRD) in children (< 24mo) [4–8]. Studies show children (0-5mo) who were not breastfed have a 165% higher risk of suffering from diarrhea and 107% higher risk of pneumonia than children who were exclusively breastfed [5, 8]. Worldwide, not breastfeeding according to recommendation is attributed to the death of 823,000 children (< 5y) and 20,000 deaths due to breast cancer each year [1].

In Indonesia, 96% of children (< 24mo) are breastfed at least once, but only 42% of children (< 6mo) are exclusively breastfed [9]. As a result, not breastfeeding according to recommendation in Indonesia is estimated to contribute to 5377 preventable infant deaths due to diarrhea and PRD per year [10, 11].

Even though Indonesia has policy level laws and regulations supporting exclusive breastfeeding [12, 13], the ineffective monitoring, implementation, alongside other institutional barriers, prevents greater progress [14]. Wide scale issues such as aggressive marketing of breast milk substitutes (BMS) and limited workplace lactation spaces for breastfeeding mothers [15, 16] are still issues as a result of a lack of monitoring and enforcement of existing regulations. Although the World Health Organization (WHO) International Code of Marketing of Breast Milk Substitutes [12, 17–21] has been accepted, it is not sufficiently enforced. Indeed, Indonesia is one of the largest contributors to the rapid increase of the growth of BMS sales in East Asia and low and middle income countries in general, only second to China [22]. The current Indonesian policy of 12 weeks of maternity leave [23] fails to meet minimum recommendation of 18 weeks set by the International Labor Organization [24]. The ideal period is six months, to enable mothers to exclusively breastfeed and adhere to WHO recommendations for exclusive breastfeeding [3].

The lack of accredited, “Baby-Friendly” breastfeeding promoting hospitals [25] and the lack of mass media support to facilitate a culture of breastfeeding [26] contribute to high levels of not breastfeeding according to recommendation. Moreover, given the diverse political and cultural setting of Indonesia, the country may benefit from a holistic approach to increase breastfeeding rates and Infant and Young Child Feeding Practices (IYCF) and among healthcare workers, doulas, religious figures, and village heads. Multi-sectoral intervention approaches can include advocacy, training, media promotion and home visits [27]. Some of these approaches have been ongoing in some areas of Indonesia and community-based training increases breastfeeding knowledge [27–29].

Breastfeeding also has the potential to save costs for parents, insurance companies, employers, and society in general (e.g. healthcare costs, infant formula costs) [30–32]. In seven countries in South East Asia, the annual treatment cost stemming from cases of childhood diseases attributed to not breastfeeding according to recommendation is large (US$293.55million), of which it was estimated that 87% of the costs came from Indonesia [10]. As Indonesia recently introduced national health insurance in 2014, these costs may be borne mostly by the government in the near future and this may put pressure on the already low health expenditure in Indonesia. The per capita healthcare expenditures adjusted for Purchase Power Parity (PPP) was US$3.02/capita in 2016 and was only slightly higher than that of India (US$2.69 PPP). Indonesia’s healthcare budget is the lowest GDP % among OECD countries at 2.8% (2016) [33].

The costs estimate attributed to not breastfeeding according to recommendation by Walters et al. [10] (including both provider and patient costs) are substantial. However, the healthcare system cost in the above-mentioned study was estimated using costs from one region in Indonesia and may not fully represent other regions in Indonesia. Therefore, the present study seeks to provide a more comprehensive national expenditure estimate by surveying five different regions in Indonesia.

This study addresses the following questions. First, using the sub-national level unit cost and utilization data for more accurate estimates, what are the health system costs of treating diarrhea and PRD for children not breastfed according to recommendation in Indonesia? Second, what is the out-of-pocket financial burden of patients’ family member(s) in accessing care?

We aim to present the economic impact of not breastfeeding according to recommendation, extrapolated by treatment costs of diarrhea and PRD among children (< 24mo) from the healthcare provider perspective and patient perspective. To our knowledge, such study in Indonesia is limited to one study [10]. Knowing the economic impact of not breastfeeding according to recommendation in Indonesia should spur policy makers and society in general into action to improve breastfeeding policies and practices.

## Methods

The methods used were based on the Walters et al. and Bagriansky study [10, 34]. Provider costs were assessed in five different categories of healthcare facilities; public hospitals, private hospitals, village health posts (*posyandu*), primary health centres (*puskesmas*), and “others” being other healthcare facilities. For hospitals, cost data was collected separately for inpatient and outpatient visits. The “others” category consists of facilities not grouped within the other five types of facilities. For these, we used the treatment cost of the most similar of the five types of institutions from our survey data. The list of other facilities, is provided in an Additional file 1. Data collection from 2015 was used to estimate the costs from Bandung city, while in the rest of locations, data collection was from 2016. All costs were converted to USD using the 2016 reference exchange rate from Bank of Indonesia [35].

Data collection was performed in five separate sites, two cities and three non-urban districts, ranging from West to near East Indonesia. The five selected sites where; Bandung city (West Java), Serdang Bedagai district (North Sumatra), Tomohon city (North Sulawesi), Gianyar district (Bali), and Kupang district (East Nusa Tenggara). The site selection was based on discussions with stakeholders and experts from the Ministry of Health, the Ministry of Children and Women Empowerment, UNICEF Indonesia, and Alive & Thrive. The treatment costs obtained from each site were used to represent similar provinces. The list of provinces is provided in Additional file 2. In total, data was collected from 13 facilities.

The Gates Reference Case principles [36] was followed in conducting and reporting our study. However, since this study only focused on the cost of not breastfeeding according to recommendation, some principles, such as calculating Disability-Adjusted-Life-Years, was not pertinent. However, whenever relevant, the appropriate case principles were applied.

### Provider perspective

Costs from the providers’ perspective consisted of medical expenditures for treatment. To calculate these expenditures, the following information related to treating diarrhea and PRD for children (< 24mo) was collected from records and interviews with healthcare workers. The labour costs, equipment costs, number of equipment and the recurrent costs of upkeep and supplies as well as service utilization (i.e. number of visits/cases) were observed. Overhead costs such as utilities, rent and administration were not included. Interviews were conducted with 26 healthcare workers, two per facility in each region, to estimate the percentage of utilization of resources to treat patients. Annualized capital costs were calculated using a 3% discount rate, in which the total cost for capital items is spread over the useable life time of the equipment and is standard for economic evaluations of healthcare programs [37]. The cost/treatment was not differentiated for the type or stage of diarrhea and PRD; therefore, these calculations do not reflect the differences in costs by different types and stage of diseases. The cost per treatment differences only stem from differences in treatment provided in different health facilities and regions.

### Patient perspective (out of pocket cost, medical and non-medical)

In total, 615 caregiver/child pairs were interviewed to estimate out of pocket costs from patient perspective (patient medical and non-medical costs). Caregivers were interviewed regarding medical costs (e.g. lab and drug costs), and non-medical costs such as transportation and productivity loss. Productivity loss was measured based on the work time lost due to accessing the facilities. The mean total time for roundtrip transport, receiving treatment in facilities (including wait time) was assessed and multiplied by the estimated wage per minute of the accompanying family member(s) to estimate productivity loss. A monetary value was not allotted for those providing unpaid childcare such as housewives or stay-at-home fathers. Also, the productivity loss or extra expenses stemming from providing outpatient homecare was not calculated. A sampling of convenience was used and 20 to 70 caregiver/child pairs from each type of facility studied were interviewed. In locations without certain types of facilities, for example Tomohon city does not have a public hospital, more participants were interviewed in the other facilities. Prior to the interviews, respondents were required to give informed signed consent.

### Total prevalence of diarrhea and PRD

**Fig. 1:**
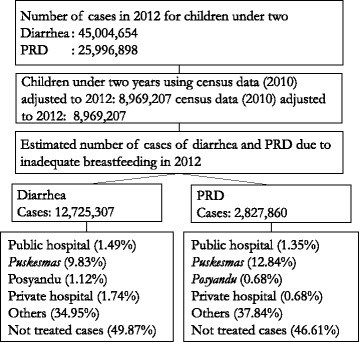
Total number of diarrhea and PRD cases per healthcare facility in Indonesia. This figure shows the number of diarrhea and PRD cases and the percentage of those cases which were treated in certain facilities

The Indonesia Demographic Health Survey (IDHS) 2012 data [9] was used to estimate the prevalence of diarrhea and PRD from each province in Indonesia, and the proportion of those seeking treatment for the country as a whole. The prevalence rates of diarrhea and PDR for children <6mo was 11.80% and 2.30% respectively. The rates for children between 6mo - 24mo for diarrhea and PRD was 20.61% and 5.39% respectively [9]. The attributable relative risk of diarrhea and PRD due to not breastfeeding according to recommendation was taken from a meta-analysis of studies including Walters’ et al. [10], Lamberti et al. [5, 8] and the Child Health Epidemiology Reference Group [38]. The relative risk ratios were then compared with the 2010 census data that sampled the total number of children (< 24mo) [39]. This figure was multiplied by 26 to estimate the number of cases per year, and, later, by the percentage of patients accessing various facilities (e.g. public or private hospitals, *puskesmas*, *posyandu*, or others) from IDHS 2012 data [9]. The total annual number of cases treated per facility was obtained and this method of calculation is presented in Fig. 1. The proportions of outpatient and inpatient visits was obtained from Indonesia Health Profile 2009 [40] as it has the most complete data set available in this respect. We assumed a similar proportion for public and private hospitals (60.85% and 91.60% for outpatient diarrhea and PRD treatment, and 39.15% and 8.40% for inpatient diarrhea and PRD treatment, respectively). The Additional file 3 presents the summary of the type of costs and parameters used for this study.

### Healthcare system cost in Indonesia

The total healthcare system costs consisted of tallying the total costs from provider and patient perspectives in treating diarrhea and PRD for children (< 24mo) due to not breastfeeding according to recommendation. Patient medical costs were excluded from the healthcare system costs analysis to avoid duplication of expenditures as these were also counted as expenditures by healthcare providers.

To calculate the total healthcare system costs, the annual number of cases were multiplied by the treatment costs throughout the respective facilities and regions (which already includes the costs from provider and patient perspectives), resulting in the total treatment cost implications of these diseases due to not breastfeeding according to recommendation.

### Sensitivity analysis

In order to present the high and low estimates of our calculation, as sensitivity analysis was preformed where the main parameters were varied by up to ±25%. The treatment cost for each facility, prevalence rate of diarrhea and PRD and the rate of accessing care by was modify by ±25%, ± 25% and ±5%, respectively.

## Results

### Participant characteristics

Participants caregiver/child pairs for those treated for diarrhea and PRD were surveyed in the different types of healthcare facilities. Participant characteristics are presented below in Table 1 with a majority of caregivers who accompanied the child were female. The mean household income of participants visiting public hospitals was US$109/mo. Participants spent a mean of 72 min on round-trip travel time and 223 min at the hospital, which was more than the other healthcare facilities surveyed. Children surveyed at the public hospital suffered either from diarrhea or both diarrhea and PRD. The mean household income of accompanying caregivers visiting private hospitals was US$103. They spent, on average, 38 min on round-trip travel and 148 min in the hospital. Most of the private hospital patients in our survey suffered from PRD.

**Table 1:** Characteristics of caregiver/child (< 24mo) pairs with diarrhea or PRD (*n* = 615) in 13 healthcare facilities in Indonesia, 2016

Items	Type of facility			
	Public hospital, total *n* = 91	Private hospital, total *n* = 107	P*uskesmas*, total *n* = 368	*Posyandu*, total *n* = 50, Bandung city
Age, mean (95% C.I)	33 (31–34)	31 (31.2–31.4)	31 (30–32)	30 (28–32)
Gender of accompanying caregiver (female)	80 (88%)	97 (91%)	362 (98%)	48 (96%)
Employed family members	63 (69%)	24 (48%)	87 (24%)	16 (32%)
Education level attained of family members (senior high school or higher)	51 (56%)	81 (76%)	200 (54%)	27 (54%)
Caregiver Marital Status (married)	88 (97%)	77 (72%)	344 (93%)	48 (96%)
Number of children (mean)	2.30	2.18	2.42	2.04
Means of transport to health facility				
Own motorcycle	26 (29%)	34 (32%)	98 (27%)	13 (26%)
Other (e.g. own car, public transport)	65 (71%)	73 (68%)	270 (73%)	37 (74%)
Journey time in minutes, mean (min - max)	72 (5–480)	38 (5–180)	14 (1–120)	13 (2–90)
Time spent in facility in minutes, mean (min - max)	223 (0–720)	143 (3–3360)	45 (1–2400)	57 (5–180)
Monthly average income, US$^a^, mean (95% CI)	109 (78–140)	103 (56–150)	31 (22–41)	56 (29–83)
Patient’s type of disease	*n = 73* ^b^		*n = 314* ^b^	
Diarrhea	26 (36%),	32 (30%)	59 (19%),	2 (4%)
PRD	12 (16%)	59 (55%)	197 (63%)	42 (84%)
Both	35 (48%)	16 (15%)	58 (18%)	6 (12%)

This table shows the accompanying family member(s) characteristics, as well as the patients’ type of diseases

^a^Exchange rate Rp 13,120/US$

^b^Due to incomplete data provided by some respondents

Participants visiting the *puskesmas* and *posyandu* had an average monthly income of US$31 and US$56, respectively. They spent about 13 min round-trip travel time to the facilities and approximately 50 min at the facilities. Most participants surveyed at the *puskesmas* or *posyandu* suffered from PRD.

### Healthcare system costs

The annual costs of outpatient and inpatient care for diarrhea and PRD treatment due to not breastfeeding according to recommendation in the five types of facilities are provided in Table 2. The mean costs from provider perspective for both diarrhea and PRD combined was US$11.37. The cost from patient perspective was US$3.85/treatment and included transportation and productivity loss due to seeking treatment.

**Table 2:** Healthcare system costs of treating diarrhea and PRD due to not breastfeeding according to recommendation in Indonesia, 2016^a^

	Public hospital		Private hospital		*Puskesmas*		*Posyandu*		Others		Total	
Items	Diarrhea	PRD	Diarrhea	PRD	Diarrhea	PRD	Diarrhea	PRD	Diarrhea	PRD	Diarrhea	PRD
*Outpatient*												
Costs per year (US$ thousands)	4769	1595	2882	357	3546	1208	36,341	43	51,204	11,295		
Cost per case treated (US$)	41.33	45.62	21.39	20.24	2.84	3.33	1.39	2.24	11.51	10.56		
*Inpatient*												
Costs per year (US$ thousands)	26,933	1176	12,666	700	n/a	n/a	n/a	n/a	n/a	n/a		
Cost per case treated (US$)	362.80	366.82	146.11	433.44	n/a	xn/a	n/a	n/a	n/a	n/a		
Total cost (000US$)											102,241	16,375
Per patient (US$)											16.27	10.85
									Annual total healthcare system costs		US$ 118.62 million (US$15.22/case treated for both diarrhea and PRD)	
									Provider perspective		US$ 88.64 million (US11.37/case treated for both diarrhea and PRD)	
									Patient perspective (non-medical cost)		US$ 29.98 million (US3.85/case treated for both diarrhea and PRD)	

This table shows the healthcare system costs of treating infants suffering from diarrhea and PRD due to not breastfeeding according to recommendation in five different facility categories. Total costs from provider perspective is also presented

^a^Exchange rate Rp 13,120/US$

**Fig. 2:**
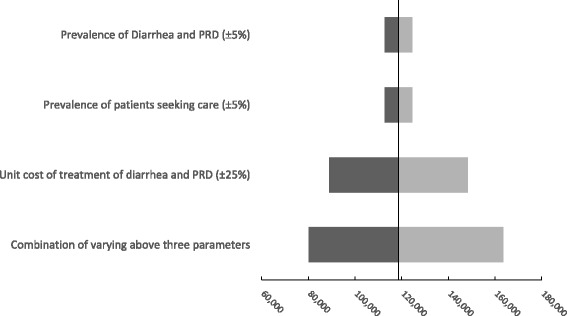
Sensitivity analysis. This figure shows the result of our sensitivity analysis for the costs estimation by varying selected main parameters. The sensitivity analysis shows that the highest estimate may reach US$163.5 million, while the lowest estimate is US$80.3 million

The highest cost per treatment was at the private hospital for both outpatient and inpatient care. For outpatient services, the highest treatment cost per patient was for inpatient PRD treatment in private hospitals. The outpatient treatment cost of diarrhea ranged from; US$1.39 in *posyandu*, US$41.33 in public hospitals and US$146.11 in a private hospital. The costs for inpatient treatment for diarrhea can reach US$362.80 in a public hospital. The cost per case treated as outpatient PRD treatment ranged from US$2.34 in *posyandu*, US$45.62 in public hospitals. PRD inpatient treatment was US$366.82 in public hospitals and US$433.44 in private hospitals. The treatment costs in each facility was higher in Bandung city, except for outpatient treatment in the public hospital in which the treatment cost in Serdang Bedagai district is around one and a half to two times higher (unpublished data available from authors). The total annual healthcare system cost was around US$119 million (provider perspective costs is approximately US$89 million, while the patient perspective costs is around US$30 million). From the patient perspective, the non-medical cost share out of the total healthcare system cost differs between diseases, in which for PRD the non-medical cost share can reach around 55% of the total healthcare system cost, while for diarrhea the non-medical cost share is 20%. Our sensitivity analysis shows that the highest estimate may reach US$163.5 million, while the lowest estimate is US$80.3 million (Fig. 2).

### Out of pocket cost (medical and non-medical, patient perspective)

Table 3 provides the patient’s accompanying family member(s) costs, separated by type of facility. This includes medical (e.g. lab and drug costs) and non-medical (e.g. transport, productivity loss) costs. Differences were mostly due to higher fees charged. The per patient costs at *puskesmas* was around 10 times lower than the private hospital. The total out of pocket cost of accessing treatment for diarrhea and PRD attributed to not breastfeeding according to recommendation from the patient perspective was estimated to be US$83 million every year.

**Table 3:** Out of pocket cost for accessing diarrhea and PRD treatment (*n* = 615) for children (< 24mo) in Indonesia^a^

	Public hospital	Private hospital	Primary health centre	Local village health post	Others
*Outpatient*					
Costs per year (000US$)	2974	3455	4237	381	65,489
Per case treated (US$)	19.78	22.68	2.63	1.97	11.87
*Inpatient*					
Costs per year (000US$)	2432	4420	n/a	n/a	n/a
Per case treated (US $)	31.41	50.05	n/a	n/a	n/a

This table shows the out of pocket costs for accessing diarrhea and PRD treatment, basically the costs from the patients’ accompanying family member(s)’ perspective

^a^Exchange rate: Rp13,120/US$, including travel, registration, lab and drugs costs, as well as productivity losses

## Discussion

These results highlight the economic impact of not breastfeeding according to recommendation through the treatment costs of diarrhea and PRD in Indonesia. The annual healthcare system cost of not breastfeeding according to recommendation associated with associated with diarrhea and PRD amounts to US$119 million per year (1.6 trillion Rupiah), which is 0.01% of Indonesian Gross National Income (GNI) in 2012 [41]. Of this, 80% of the expenses comes from treating diarrhea (Table 2). From the provider perspective, the average treatment cost per case of both diarrhea and PRD is US$11.37, which is 10.6% of annual healthcare expenditure per capita of Indonesia in 2012 [42]. Translating these average treatment costs using 2016 purchase power parity figs. [43], we found that these treatment costs are lower than that of Laos, Thailand, and Viet Nam, roughly similar to Timor Leste, and around two times higher than that of Cambodia and Myanmar [10, 34, 44, 45]. Regardless, a significant share of these expenditures could be translated into cost-savings preventative measures by the Ministry of Health, and reallocated towards other priorities such as IYCF and breastfeeding promotion interventions. It is important to note, however, that the study by Walters et al. has shown that the highest non-medical costs of not breastfeeding according to recommendation in Indonesia came from the long-term impact of cognitive losses, amounting to US$1344 million per year. This was the largest impact when compared to the other countries in the study [10]. Moreover, the same study also showed that the amount of child and maternal death (from breast cancer) attributed to not breastfeeding according to recommendation was also the largest in Indonesia, amounting to more than 5000 and 2000 deaths respectively. Including these impacts to our current study would further highlight the egregious cost of not breastfeeding according to recommendation to the society, not to mention the further impact caused by the cognitive loss and death.

Secondly, the costs of treating diarrhea and PRD for children (< 24mo) amounted to approximately US$88 million from provider perspective (i.e. excluding patient perspective costs), and accounts for 75% of the total healthcare system costs. This leaves around 25% of the healthcare system costs to come from non-medical costs borne by patients (i.e. transport, productivity loss), amounting to around US$30 million per year (US$3.85 per case). Differentiating between diarrhea and PRD, we found that this percentage increased substantially for PRD (55%, US$6.00 per case), while the percentage is at 20% for diarrhea (US$3.33 per case). This shows that although overall the costs of diarrhea are higher than that of PRD due to higher number of cases, the latter cause much more burden to the patients through non-medical costs. Furthermore, PRD has a higher mortality rate in Indonesia [10]. Note that we did not put monetary value to caregivers for unpaid work at home. Therefore, the figures underestimated the financial burden on homemakers and stay-at-home fathers, and on providing outpatient care for the patients and palliative care. Therefore, these results undervalue the true productivity loss and, subsequently, the societal cost of diarrhea and PRD due to not breastfeeding according to recommendation. These costs may potentially be avoided if breastfeeding according to recommendation was done properly.

Thirdly, the patient out of pocket costs (including medical and non-medical costs) of accessing both outpatient and inpatient care for diarrhea and PRD treatment are a potential problem for families as they account for more than 10% of average monthly income [46], and much higher in the case of inpatient care. Costs requiring this much of the average income may cause patients to avoid visiting the facilities for treatment which may lead to more severe diarrhea and PRD cases, and may, to some extent, explains the almost 50% of untreated cases in our finding as it is a significant barrier to access treatment. In this context, providing breastfeeding according to recommendation to prevent diarrhea and PRD [4–8] would be beneficial to patients’ family members as they can save financial resources. Be mindful that our productivity loss calculation may undervalue the true cost occurring as previously explained. In addition, not breastfeeding according to recommendation may necessitate purchasing BMS for infants which may further increase family expenses. Both breast milk substitute expenses and medical expenses due to diarrhea and PRD have been shown in other studies in the Philippines [47, 48]. Even though our study does not explore the cost of purchasing BMS and the subsequent medical expenses, these concerns should be addressed as it does affect the financial impact on families and may dictate whether they feel they can afford to seek medical attention or not.

To reduce the incidence of not breastfeeding according to recommendation the following recommendations are proposed. The National Health Insurance policy (NHI) or “*Jaminan Kesehatan Nasional”,* can be directed towards covering the costs of counselling on infant and young child feeding, including breastfeeding, information which is accessible to all mothers, therefore, potentially decreasing the treatment costs of diseases associated with not breastfeeding according to recommendation. Currently, the NHI is focused mostly on the curative side, and not so much on the preventive aspect. Therefore, there might be a need to revise NHI to cover more preventive and public health programs and interventions such as the counselling on breastfeeding to all mothers. The NHI may become one of the solutions to provide the appropriate funding to cover the costs of public health programs and interventions to ensure the success of breastfeeding. Thus, eliminating the incidence of diseases caused by not breastfeeding according to recommendation. Prioritizing resources to support breastfeeding according to recommendation is therefore crucial, with a strong return on investment for the entire nation.

In addition, a comprehensive breastfeeding strategy should be implemented to ensure breastfeeding is promoted, protected, and supported. The role of workplaces, healthcare workers, and social support from families cannot be underestimated. Creating an enabling environment for breastfeeding is crucial, and all stakeholders play significant roles. Providing longer maternity leave and supporting women to continue breastfeeding after returning to work are just two examples of how environments can be designed to support breastfeeding according to recommendation [49–51]. Moreover, six months of maternity leave alone is insufficient to ensure exclusive breastfeeding if mothers do not have access to skilled counseling support and if social norms around breastfeeding are not supportive. The World Bank estimated that scaling up a core comprehensive breastfeeding strategy to achieve the Global Nutrition Target for breastfeeding could generate a return on investment of US$35 dollars for every US$1 invested across all low-and middle-income countries [11]. With its growing economy and demand for a skilled workforce, Indonesia can benefit economically from a similar investment in interventions to protect, promote, and support breastfeeding.

In terms of study limitations, Indonesia is an extremely diverse nation, and a broader sampling of treatment costs is needed for more accurate results. Data regarding treatment costs were assessed from two cities and three districts to represented the treatment cost of 98 cities, 416 districts and 34 provinces in Indonesia. Furthermore, our study sample to calculate patient costs is relatively small (615 respondents) in the context of the number of people in Indonesia (more than 250 million people). However, we found that patients per type of facility in each of the city/districts have similar trends in terms of distance/time travelled, and monthly income/expenditure. Therefore, as our selection of cities/districts is spread in five different parts of Indonesia, we believe that our sample, to some extent, may represent the trend in Indonesia. Future studies can explore treatment and patient costs from additional regions, so more islands or regions in Indonesia will be represented by the extrapolations of data gathered here.

Another limitation was the assumption that the relative risk of diarrhea and PRD due to not breastfeeding according to recommendation was evenly distributed across Indonesia [10]. Further study on calculating this relative risk specifically for each region in Indonesia is required, as there is a high possibility that variation is high. This, in turn, may yield better cost estimation.

Lastly, the productivity value of housewives and stay-at-home dads were not estimated for the productivity loss and extra expenses due to providing outpatient care. Therefore, to some extent, this undervalues our calculation of non-medical cost. Future study may include these non-medical costs, and may go even further to estimate the costs of palliative care and future productivity loss of children and maternal breast cancer deaths attributable to not breastfeeding according to recommendation and provide a more complete picture of the severe impact of not breastfeeding according to recommendation in Indonesia.

Although our study is based on the methodology used by Walters et al. [10], our study results differ as the mean treatment cost for both diarrhea and PRD combined was US$15.22 versus US$21.10 as found by Walters et al. One of the main reasons for this was the Walters et al. assessed treatment cost from only Bandung city, whereas in this study we included data from one additional city and three districts in different regions to represent the costs from different provinces. Provincial figures of different diarrhea and PRD prevalence was also used. Our study found that costs were generally higher in Bandung city than other regions. Therefore, our results may better represent the cost differences throughout Indonesia, and includes a greater emphasis on lower cost regions/locations.

## Conclusion

The 2016 annual economic impact of not breastfeeding according to recommendation in Indonesia based on the high cost of treating diarrhea and PRD warrants urgent attention. The healthcare system costs and non-medical out of pocket cost were US$11.37 and US$3.85/treatment respectively. The total healthcare system costs were US$118.62 million annually. Non-medical costs from patient perspective accounted for 25% of treatment costs. Out of pocket costs represented a significant burden and a financial barrier to seeking treatment as it accounts for more than 10% of the caregiver’s average monthly income.

Importantly, these figures only include immediate costs stemming from not breastfeeding according to recommendation and do not take into account the cost of future cognitive losses, which could reach US$1.54 billion per year for Indonesia [10].

## Additional files

Additional file 1. Classification of health facilities and proxy for treatment cost (based on IDHS 2012). This table shows the proxy of treatment cost used for facilities included within “others” category. (DOCX 15 kb)

Additional file 2. Proxy for treatment costs in different provinces. This table shows the proxy of treatment cost used to represent each of the provinces in Indonesia. (DOCX 15 kb)

Additional file 3. Summary of type of costs/parameters used. This table shows the summary of explanation, values, and sources of various costs and parameters used in the study. (DOCX 20 kb)

## Abbreviations

BMS: Breast milk substitutes
GNI: Gross National Income
HCW: Healthcare worker
IDHS: Indonesia Demographic Health Survey
NHI: National Health Insurance policy
OECD: Organisation for Economic Co-operation and Development
*Posyandu*: Health post
PPP: Purchasing Power Parity
PRD: Pneumonia/respiratory disease
*Puskesmas*: Primary health centre
UNICEF: United Nations International Children’s Emergency Fund
WHO: World Health Organization
