# Supplementary material for: The annual cost of not breastfeeding in Indonesia: the economic burden of treating diarrhea and respiratory disease among children (< 24mo) due to not breastfeeding according to recommendation
Source: Int Breastfeed J. 2018 Mar 2;13:10. doi: 10.1186/s13006-018-0152-2 (PMC5833067; doi:10.1186/s13006-018-0152-2)
Supplement: Supplementary file 1 — Classification of health facilities and proxy for treatment cost (based on IDHS 2012). This table shows the proxy of treatment cost used for facilities included within “others” category. (DOCX 15 kb) [file 13006_2018_152_MOESM1_ESM.docx]

| **Additional file 1. Classification of health facilities and proxy for unit cost (based on IDHS 2012)** | | |
| --- | --- | --- |
| **Type of facility** |  | **Classification and proxy for unit cost** |
| Government hospital |  | Calculated |
| Community/primary health center (*puskesmas*) |  | Calculated |
| Public clinic |  | *Puskesmas* |
| Village health post (categorized as *posyandu*) |  | *Posyandu* |
| Public delivery post (categorized as *posyandu*) |  | *Posyandu* |
| Public health post (*posyandu*) |  | Calculated |
| Public other |  | *Posyandu* |
| Community specific public sector |  | *Puskesmas* |
| Private hospital |  | Calculated |
| Private pharmacy |  | Private hospital outpatient |
| Private doctor |  | Private hospital outpatient |
| Private maternity hospital (categorized as private hospital) |  | Private hospital outpatient and inpatient |
| Private maternity home |  | Private hospital outpatient |
| Private clinic |  | Private hospital outpatient |
| Private pediatrician |  | Private hospital outpatient |
| Private midwife |  | *Puskesmas* |
| Private nurse |  | *Puskesmas* |
| Private village midwife |  | *Puskesmas* |
| Private other |  | *Puskesmas* |
| Traditional birth attendant |  | *Posyandu* |
| Private shop |  | Private hospital outpatient |
| Community specific other sector |  | *Puskesmas* |
| Other |  | *Puskesmas* |
|  | | |
