# Supplementary material for: The annual cost of not breastfeeding in Indonesia: the economic burden of treating diarrhea and respiratory disease among children (< 24mo) due to not breastfeeding according to recommendation
Source: Int Breastfeed J. 2018 Mar 2;13:10. doi: 10.1186/s13006-018-0152-2 (PMC5833067; doi:10.1186/s13006-018-0152-2)
Supplement: Supplementary file 2 — Proxy for treatment costs in different provinces. This table shows the proxy of treatment cost used to represent each of the provinces in Indonesia. (DOCX 15 kb) [file 13006_2018_152_MOESM2_ESM.docx]

| **Additional file 2. Proxy for unit costs in different provinces** | | |
| --- | --- | --- |
| **Province** |  | **Unit cost used** |
| Aceh  North Sumatera  West Sumatera  Riau  Jambi  South Sumatera  Bengkulu  Lampung  Bangka Belitung Isle  Riau Isle |  | Serdang Bedagai District (North Sumatra) |
|  |  |  |
| Jakarta (Capital Region)  West Java (including the now Banten province)  Central Java  Jogjakarta (Special Region)  East Java  Banten |  | Bandung City (West Java) |
|  |  |  |
| Bali  West Borneo  Central Borneo  South Borneo  East Borneo |  | Gianyar District (Bali) |
| West Nusa Tenggara  East Nusa Tenggara  West Papua  Papua  Mollucas  North Mollucas |  | Kupang District (East Nusa Tenggara) |
|  |  |  |
| North Celebes  Central Celebes  South Celebes  South East Celebes  Gorontalo  West Celebes |  | Tomohon City (North Sulawesi) |
| *We excluded Banten province since it is a newly established province (previously part of West Java province) and was not yet included in the 2010 census data, therefore we cannot include it in our calculation. Within the 2010 census data, the population of Banten province is still calculated within West Java province. | | |
