# Supplementary material for: The annual cost of not breastfeeding in Indonesia: the economic burden of treating diarrhea and respiratory disease among children (< 24mo) due to not breastfeeding according to recommendation
Source: Int Breastfeed J. 2018 Mar 2;13:10. doi: 10.1186/s13006-018-0152-2 (PMC5833067; doi:10.1186/s13006-018-0152-2)
Supplement: Supplementary file 3 — Summary of type of costs/parameters used. This table shows the summary of explanation, values, and sources of various costs and parameters used in the study. (DOCX 20 kb) [file 13006_2018_152_MOESM3_ESM.docx]

| **Additional file 3. Summary of type of costs/parameters used** | | | | |
| --- | --- | --- | --- | --- |
| Items |  | Type of cost/parameters used |  | Values/sources |
| Provider perspective costs |  | Recurrent resources used for treatment (e.g. medicine, syringe) |  | Patient billing data, hospital record on resource utilization, interview with HCW |
|  |  | Capital resources used for treatment (e.g. equipment, furniture) |  |  |
|  |  | Costs of capital and recurrent resources |  | Patient billing data, market price |
|  |  | Discount rate |  | 3% [38] |
|  |  |  |  |  |
| Patient perspective costs* |  | Time spent accessing treatment (two ways transport time, waiting time, time spent receiving treatment, time spent as inpatient) |  | Interview with patients’ family member(s) |
|  |  | Administration/registration cost |  |  |
|  |  | Treatment related cost (e.g. lab cost, medicine) |  |  |
|  |  | Non-medical costs (i.e. productivity loss, transport cots) |  |  |
|  |  | Wage/expenditure per month (to estimate productivity) |  |  |
|  |  | Time of work per week (days) |  |  |
|  |  | Type of transport used |  |  |
|  |  |  |  |  |
| Healthcare system costs |  | Prevalence rate [9] |  |  |
|  |  | Diarrhea |  | 11.80% (under 6 months), 20.61% (6 – 23 months) |
|  |  | PRD |  | 2.30% (under 6 months), 5.39% (6 – 23 months) |
|  |  | Population attributable risk of diarrhea and PRD due to not BAR [5,8,10,39] |  |  |
|  |  | Diarrhea |  | 25.85% |
|  |  | PRD |  | 39.33% |
|  |  | Proportion of inpatient and outpatient for those visiting public and private hospitals [41] |  |  |
|  |  | Diarrhea |  | 60.85% outpatient, 39.15% inpatient |
|  |  | PRD |  | 91.60% outpatient, 8.40% inpatient |
|  |  |  |  |  |
| * information obtained from and comprises of all patients’ family member(s)accompanying patients | | | | |
